# Firearms Are Now the Leading Cause of Traumatic Brain Injury–Related Mortality in Children

**DOI:** 10.1089/neur.2023.0069

**Published:** 2023-11-23

**Authors:** Samantha R. Neuman, Cordelia Mannix, Rebekah Mannix

**Affiliations:** ^1^ Lehigh University, Bethlehem, Pennsylvania, USA.; ^2^ Middlesex School, Concord, Massachusetts, USA.; ^3^ Division of Emergency Medicine, Boston Children's Hospital, Boston, Massachusetts, USA.

**Keywords:** epidemiology, firearm violence, pediatrics, traumatic brain injury

## Abstract

Recent studies have demonstrated a significant change in the epidemiology of injury fatalities in children, most notably a marked increase in firearm-related deaths. Few studies have specifically addressed pediatric TBI-related mortality trends. Studying these trends is important for both clinical preparedness and public health interventions. The purpose of this study therefore is to examine recent trends in mechanisms, intents, and rates of pediatric TBI fatalities. Data regarding fatalities from TBI for children <18 years of age from 2011 to 2021 were extracted from the Centers for Disease Control and Prevention, National Center for Health Statistics' web-based injury statistics query and reporting system. We found that firearms became the leading cause of TBI fatalities in children by 2021, most frequently attributable to self-harm. Taken together, the findings from this study underscore the importance in monitoring the changing epidemiology of pediatric TBI fatalities.

## Introduction

Over the past two decades, significant progress has been made in reducing trauma-related mortality in children, thanks to the implementation of preventive measures such as seatbelt legislation for motor-vehicle passengers and helmet use in bicycling and youth sports.^1,2^ These measures have contributed to a decline in overall unintentional injury-related deaths in children.^3^ However, despite this progress, recent studies have highlighted a concerning increase in firearm-related fatalities among children 1–19 years of age.^4^

Whether or not the changes in the overall epidemiology of pediatric injury-related fatalities are also reflected in changes in traumatic brain injury (TBI)-related fatalities in children has not been described. In fact, few recent studies have specifically addressed mortality trends related to TBI in children. Studying changes in pediatric TBI fatalities is crucial for informing public health policies, identifying high-risk populations, evaluating prevention strategies, allocating resources effectively, and promoting awareness and education.

Therefore, the objective of this study is to investigate recent trends in mechanisms, intents, and rates of pediatric TBI fatalities. We therefore conducted a longitudinal analysis to examine changes in TBI mortality among U.S. children and adolescents ages 0–18 from 2011 to 2021.

## Methods

Data regarding fatalities from TBI for children ≤18 years of age from 2011 to 2021 were extracted from the Centers for Disease Control and Prevention (CDC), National Center for Health Statistics' (NCHS) web-based injury statistics query and reporting system (WISQARS). The CDC's WISQARS is an interactive, online database that provides fatal and non-fatal injury, violent death, and cost-of-injury data. Data are publicly available and provide no personal identifiable information. The study was exempt from the institutional review board.

Fatal injury data in WISQARS are based on death certificates from the national vital statistics system. From 2011 to 2015, cause of death was coded in accordance with the International Classification of Disease, 9th Revision, whereas International Classification of Disease, 10th Revision codes were used to derive cause-of-death codes from 2015 to 2021. The denominator to calculate mortality rates was based on population data from the U.S. Census Bureau. WISQARS provides death counts and death rates for the United States by demographic and injury-related factors, including age, intent, and mechanism.

Simple descriptive statistics were used for comparison of mechanisms by age and intent. Line graphs were plotted to demonstrate mortality changes over time by mechanism. Linear regressions were used to evaluate time trends in fatal firearm versus fatal non-firearm TBI fatality rates. All analyses were performed using GraphPad Prism software (version 10.0.0 for Mac; GraphPad Software, Boston, MA).

## Results

**FIG. 1.**
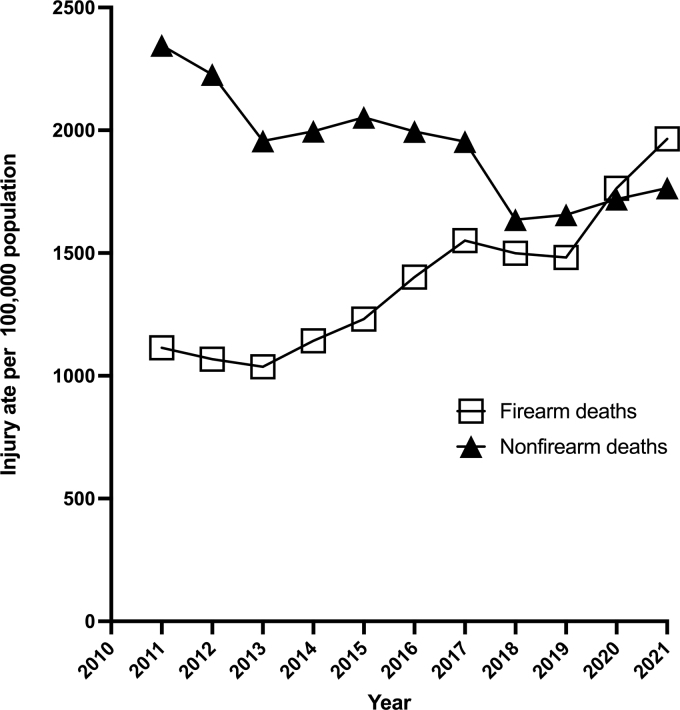
Trends in firearm and non-firearm TBI deaths. Whereas there was no change in the total number of TBI related deaths in children from 2011 to 2021 (*p* = 0.24 for trend), the total number of TBI deaths attributable to firearms increased from 1114 in 2011 to 1965 in 2021 (*p* < 0.001). In contrast, the total number of deaths from non-firearm mechanisms decreased from 2346 in 2011 to 1765 in 2021 (*p* < 0.001).

Over the study period, there were a total of 36,559 TBI-related deaths, among which 15,256 (41.2%) were attributable to firearms. The percent of total deaths from firearms increased from 32.2% in 2011 to 52.7% in 2021 (Fig. 1).

**FIG. 2.**
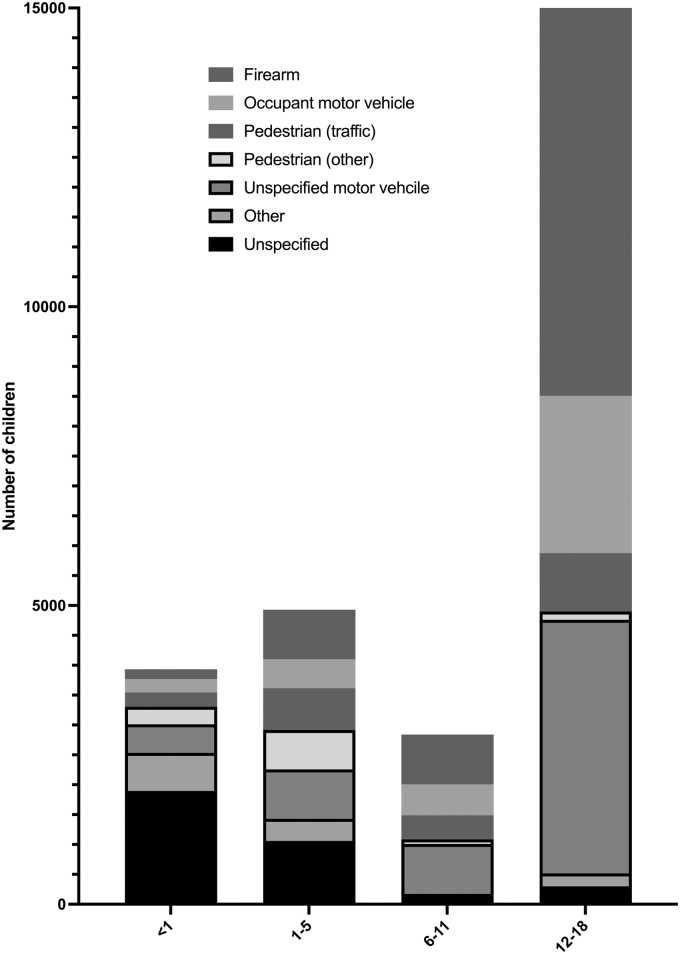
Mechanism of mortality. The majority of TBI fatalities in children occurred in adolescents 12–18 years of age. The mechanism of TBI-mortality varied by age (*p* < 0.001), with 61% of TBI-related fatalities in adolescents attributable to firearms.

**FIG. 3.**
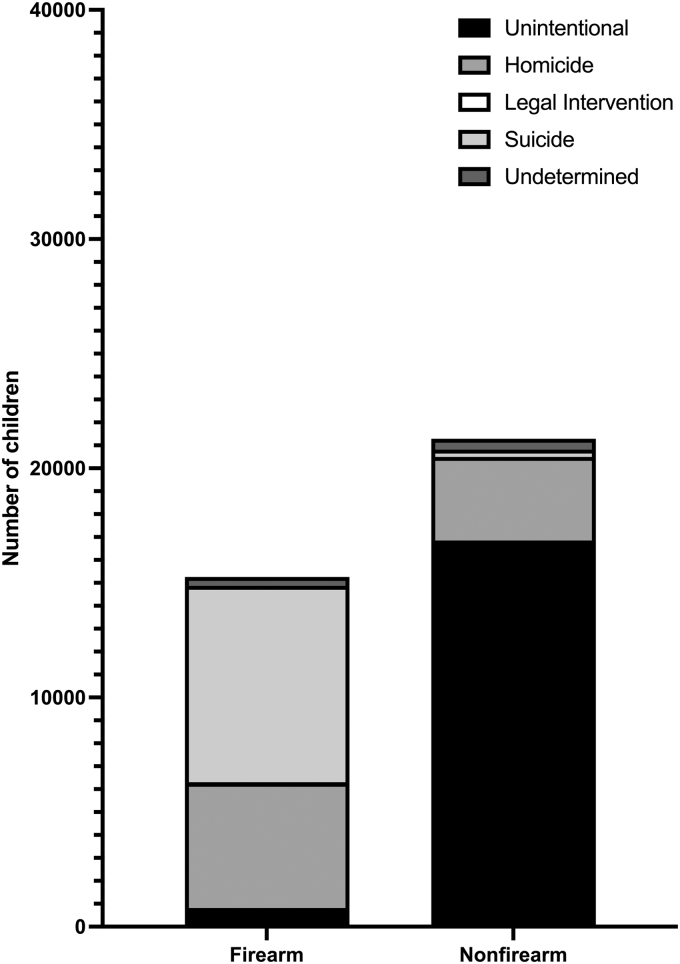
TBI mortality by mechanism and intent. Intent varied by firearm versus non-firearm mechanisms (*p* < 0.001). Most non-firearm deaths were unintentional, whereas suicides and homicides accounted for most firearm mortalities.

The mechanism of TBI-mortality varied by age. TBI mortality attributable to firearms increased with increasing age (0–1 years = 4.1%, 1–5 years = 16.8%, 6–11 years = 29.3%, and 12–18 years = 61.3% of TBI-related deaths; Fig. 2). Most non-firearm deaths were unintentional, whereas suicides and homicides accounted for most firearm mortalities (Fig. 3). The majority of non-firearm mortalities were unintentional, with 902,072 years of life lost (76.8% of total life years lost). The majority of firearm mortalities were attributable to suicide or homicide, with 696,429 years of life lost (91.4% of total).

## Conclusion

Here, we demonstrate a change in the epidemiology of TBI-related fatalities in children and adolescents, with the majority of pediatric TBI fatalities now attributable to firearms. Though past studies have shown the changing epidemiology of injury-related fatalities in children, no recent study has explored this epidemiology specific to TBI. These data have implications at both the clinical level (e.g., the training pediatric emergency medicine providers, neurosurgeons, and intensivists) as well as at the policy level (legislation mitigating children's access to firearms).

Not surprisingly, the mechanism of TBI mortality varied significantly across different age groups, with the percentage of TBI-related deaths attributed to firearms reaching 61.3% for adolescents 12–18 years of age. Notably, >15% of TBI-related fatalities in children ages 1–5 years are attributable to firearms. Child access prevention laws have previously been shown to reduce firearm-related fatalities in children, though their effect specifically on firearm-related TBI fatalities has not been studied.^5^

It is also important to note that the majority of firearm TBI-related fatalities in children occurred in the context of intentional self-harm. Measures to promote responsible gun ownership, safe storage practices, and comprehensive background checks can potentially reduce the incidence of firearm-related suicide fatalities. Additionally, increasing access to mental health services, reducing stigma, and promoting mental health awareness are vital steps in preventing self-inflicted injuries.

Taken together, the findings from this study underscore the importance in monitoring the changing epidemiology of pediatric TBI fatalities. Given the rise of firearm-related fatalities, further work is needed to identify risk factors, prevention strategies, and effective interventions to mitigate TBI-related deaths in children. Collaborative efforts from policy makers, public health professionals, and communities are crucial in addressing this significant public health issue.

## Abbreviations Used

CDC: Centers for Disease Control and Prevention
NCHS: National Center for Health Statistics
TBI: traumatic brain injury
WISQARS: Web-based Injury Statistics Query and Reporting System
